# Strategies to overcome the main challenges of the use of exosomes as drug carrier for cancer therapy

**DOI:** 10.1186/s12935-022-02743-3

**Published:** 2022-10-18

**Authors:** Bashdar Mahmud Hussen, Goran Sedeeq Hama Faraj, Mohammad Fatih Rasul, Hazha Jamal Hidayat, Abbas Salihi, Aria Baniahmad, Mohammad Taheri, Soudeh Ghafouri-Frad

**Affiliations:** 1 Department of Pharmacognosy, College of Pharmacy, Hawler Medical University, Erbil, Kurdistan Region Iraq; 2 Center of Research and Strategic Studies, Lebanese French University, Erbil, Iraq; 3 College of Medicine, Department of Medical Laboratory Sciences, Komar University of Science and Technology, Sulaymaniyah, Iraq; 4 Department of Pharmaceutical Basic Science, Faculty of Pharmacy, Tishk International University, Erbil, Kurdistan Region Iraq; 5 Department of Biology, College of Education, Salahaddin University, Erbil, Kurdistan Region Iraq; 6 Department of Biology, College of Science, Salahaddin University, Erbil, Kurdistan Region Iraq; 7 Institute of Human Genetics, Jena University Hospital, Jena, Germany; 8 Urology and Nephrology Research Center, Shahid Beheshti University of Medical Sciences, Tehran, Iran; 9 Department of Medical Genetics,, School of Medicine, Shahid Beheshti University of Medical Sciences, Tehran, Iran

**Keywords:** Cancer therapy, Exosome, Drug carrier, Exosomal delivery challenges

## Abstract

Exosomes are naturally occurring nanosized particles that aid intercellular communication by transmitting biological information between cells. Exosomes have therapeutic efficacy that can transfer their contents between cells as natural carriers. In addition, the exosomal contents delivered to the recipient pathological cells significantly inhibit cancer progression. However, exosome-based tumor treatments are inadequately precise or successful, and various challenges should be adequately overcome. Here, we discuss the significant challenges that exosomes face as drug carriers used for therapeutic targets and strategies for overcoming these challenges in order to promote this new incoming drug carrier further and improve future clinical outcomes. We also present techniques for overcoming these challenges.

## Introduction

Cancer is a critical public health concern and the world's leading cause of death, with rates increasing significantly. The most common cancer treatments are surgery, chemotherapy, radiation, and immunotherapy [1]. However, chemotherapy and/or radiotherapy are the most active and significant cancer treatments, but they cause adverse effects, drug resistance, and long-term consequences [2, 3]. Interestingly, oncology drug development targets these challenges by deploying a new cancer therapies strategy and is gathering momentum due to recent advances in drug screening technologies [4, 5]. As a cutting-edge delivery system for bioactive compounds, exosomal delivery is one of the most effective ways to deliver cancer therapies between cells.

Exosomes are naturally occurring nanoparticles that aid in intercellular communication by transmitting biological information between cells. Exosomes have recently been suggested as innovative drug delivery strategies because of their unique ability to transport particular compounds and surface proteins [6]. Furthermore, exosomes have been shown to have a function in every stage of cancer progression via mediating intercellular communication.

Intercellular communication is necessary for cells in order to respond and adjust to intracellular and extracellular changes during embryogenesis and other responses for the maintenance of the body’s homeostasis [7]. The communication mechanism by which cells communicate differs from cell to cell, ranging from direct contact to long-range interactions. Exosomes and the circulation of cell membrane particles are two mechanisms that are usually believed to be a distinct and ubiquitous system of biological signal transmits [12].

Exosomes are made when endosomes fold inward to make internal buds, which are then turned into multivesicular bodies. On the other hand, non-exosomal extracellular bodies are made directly through the budding of cell membranes. The cell that produces exosomes loads them with information in the form of proteins, nucleic acids, and lipids. This information can then significantly affect the activity of the recipient cells when the exosomes arrive at their target [8].

Recent studies showed that exosomes could be used as therapeutic tools to treat a wide range of diseases, such as cancer, as they can be loaded with both small compounds and macromolecules [9, 10]. Advances in exosome immunotherapy have demonstrated that it is a practical and safe approach that triggers both innate and adaptive immune responses. Exosomes’ distinct features open the door to new diagnostic and therapeutic possibilities. Exosomal composition, biogenesis, and releasing processes will help researchers understand and discover new cancer therapeutic strategies. Because of many challenges that have arisen, progress in the use of exosomes as drug carriers in clinical studies has been slow. Here, we describe the primary challenges that exosomes faced as drug carriers while they were being taken advantage of for therapeutic cancer objectives, as well as strategies for resolving these challenges in order to promote this new incoming drug carrier and improve future clinical outcomes. In addition, we also present interesting new techniques for overcoming these challenges.

## Innovative advances in exosome-based cancer therapy

**Fig. 1:**
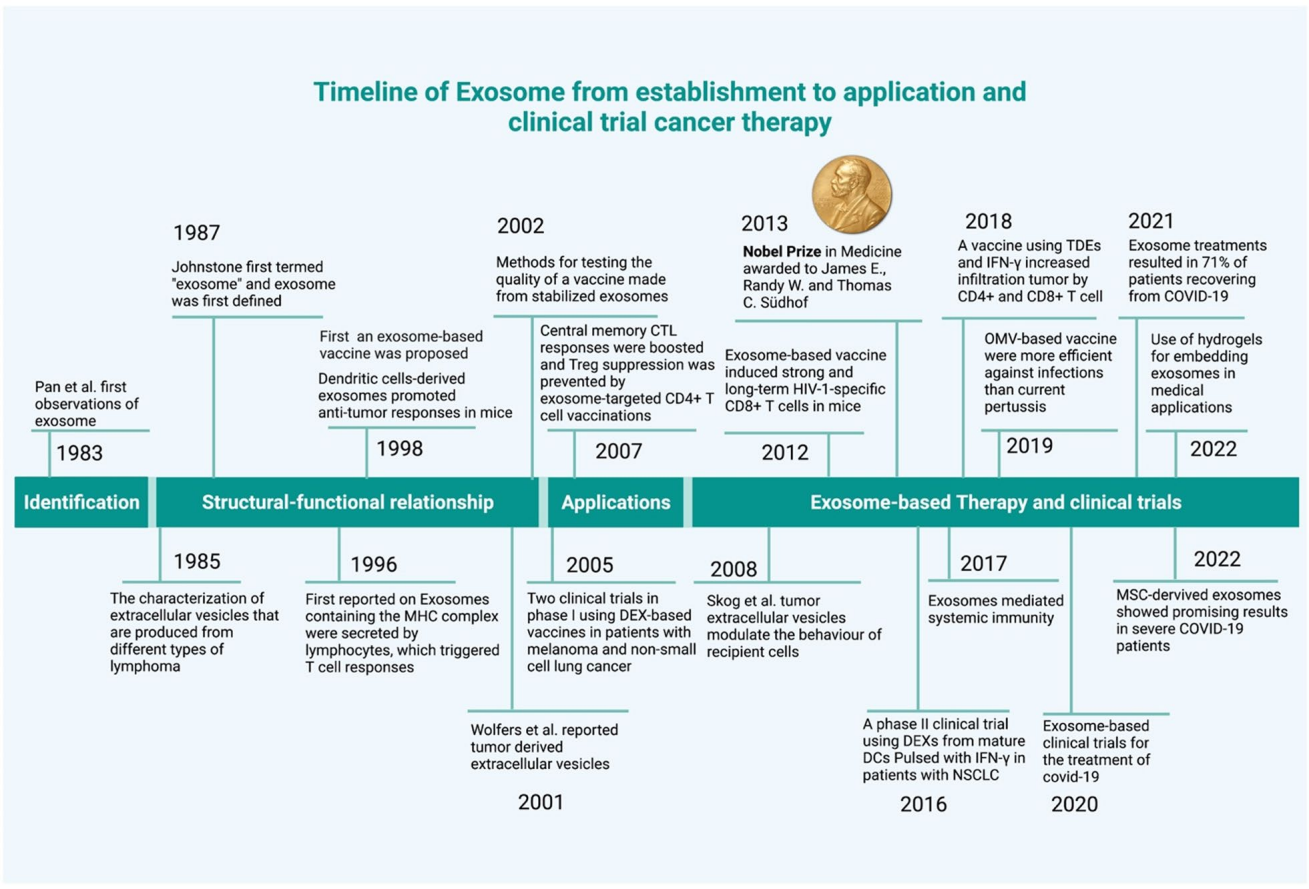
A timeline outlining the most significant findings made in relation to exosome-based therapy

Extracellular vesicles (EVs) release was first assumed to be a random event. In 1983, two separate investigations utilizing distinct animal models found that reticulocytes released transferrin receptors into EVs [11, 12]. Different lymphoma variations can manufacture EVs with diverse protein and lipid profiles, as shown by Barz and colleagues, and these EVs could be linked to tumor immune evasion and cancer spread [13]. Exosomes generated from tumor cells (TDEs) express identical antigens to TDEs, according to Schirrmacher and Barz, who discovered this in the year after their discovery [14]. The word "exosomes" was first used in 1987 by Johnstone et al. to describe EVs that express transferrin receptors [15]. After a decade, Raposo and colleagues showed the importance of exosomes in antigen presentation cells by binding MHC class II molecules in exosomes produced by B cells [16]. These results indicate that exosomes may use as biomarkers and may be applied in immunotherapeutic techniques for the development of new drugs. Several studies between the 1980s and 1990s reported on EV differential expressions, which demonstrated changed EV quantities in disease. Then, in 1998, Zitvogel et al. discovered that DEXs (exosomes derived from DCs) express MHC class I and II molecules that are functional [17]. They demonstrated that Dendritic cells (DCs) secrete antigen-presenting vesicles activated by tumor peptides and release DEXs with tumor antigens on the surface [18, 19]. This caused CTLs to help curb of tumors. Later, Wolfers and his team found that exosomes are a population of microscopic membrane vesicles that are released by cancer cells. Dendritic cells (DCs) receive tumor antigens from exosomes, which they then pass on to other cells. Following the ingestion of mouse tumor exosomes, DCs induce significant CD8+ T-cell-dependent anticancer effects on both syngeneic and allogeneic mice cancers [20]. In 2004, the Zitvogel group used an in vitro method and an animal model to explain how class I MHC molecules move from DEXs to naive Dendritic cells to stimulate CTLs effectively [21, 22]. They also explained that toll-like receptors and DEXs trigger CD8+ T cells’ MHC-restricted responses. Exosome research grew from this point on as more advanced techniques, and it became possible to do things such as construct exosomes for use in medication delivery systems and create artificial models of antigen presentation (Fig. 1).

Recent research has led to new ideas for how to treat cancer with therapeutic delivery systems based on cell-derived exosomes [23, 24]. Autologous EVs produced from patient dendritic cells were the first to be tested in clinical trials as treatments, and both phase I and phase II/III studies showed that EVs could increase the immune response to lung cancer [25–27]. Several potential clinical trials based on exosomes that are derived from autologous EVs are currently in the developing stages. Even though there are still challenges, the diagnostic and therapeutic potential of EVs is starting to be unlocked, and there is a lot of excitement about the translational uses in the next decade.

## Biogenesis and secretion of exosomes

Exosomes are generated on request from late endosomes, which originate from the internal budding of the narrowed multivesicular body (MVB) membrane, and this process results in the formation of exosomes. Intraluminal vesicles (ILVs) are made inside large multivesicular bodies (MVBs) when late endosomal membranes bulge outward [28]. Numerous proteins are inserted into the invaginating membrane during this process. At the same time, the components of the cytosol are engulfed and stored within the ILVs. After fusing with the plasma membrane, the majority of ILVs are then released into the extracellular space, where they are called "exosomes" [29]. Exosome biogenesis is regulated by a number of factors, including cell receptors and other signaling pathways. Initial endocytic vesicles are fused using caveolin-dependent or caveolin-independent mechanisms, which is the first step in developing early endosomes [30–32].

**Fig. 2:**
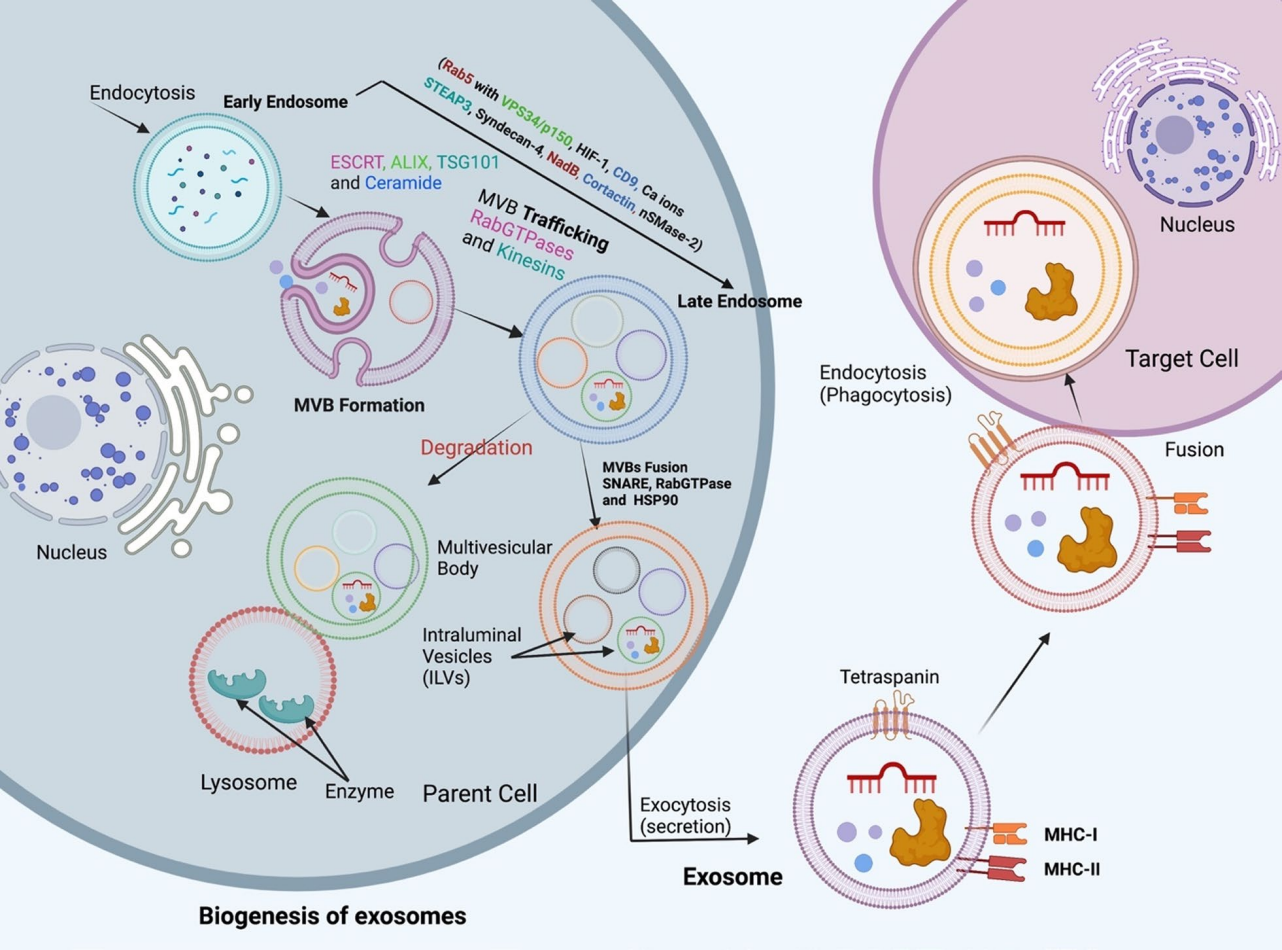
Biogenesis of exosomes shows the role of endosome in exosome formation from early endosome by invagination to late endosome and multivesicular bodies (MVBs) that contain intraluminal vesicles (ILVs). On the other hand, it shows the action of Rab5, other proteins, and molecules in exosome formation and its transport to fuse with the cell membrane and eventually release exosomes from the parent cell to the target cell

Additionally, Rab5 and its effector VPS34/p150 show their role in converting extracellular vesicles to late endosomes at the cytoplasmic membrane. Exosomes are also made by a system called the endosomal sorting complex required for transportation (ESCRT), which is in charge of sorting proteins and making ILV [33]. Each of the four ESCRT protein complexes (ESCRT-0, I, II, and III) and its related proteins, such as (VPS4, ALG-2 interacting protein X [ALIX]), participates in the formation of this machinery, which is mainly composed of over 20 proteins (Fig. 2)[34].

ESCRT plays an essential role in exosome biogenesis which mediates ILV formation, according to various studies. Exosome secretion is inhibited in various cell types, including dendritic cells and tumor cells, when Hrs, ESCRT-0 subunit STAM1, and Tsg-101 are inhibited [35, 36]. Exosome release is increased by the hormone leptin, which controls energy balance as well as hunger since leptin causes an increase in the expression of TSG-101 [37].

Components of the ESCRT, such as TSG101 and ALIX, are examples of exosome constituent proteins commonly found [38]. An accessory protein called ALIX has been shown to play a crucial role in the formation and release of exosomes. This is especially true in tumor cells, where it is essential for the construction of exosomes. For ILV assembly and consequent exosome production, ALIX interacts with syndecan heparan sulfate proteoglycan through its cytoplasmic adaptor syntenin [39]. The interaction between ALIX and syndecan affects the sorting of syndecan interactive payloads into ILVs [40]. Additionally, ESCRT-III is recruited directly to late endosomes by ALIX, making tetraspanin integration and secretion into the exosomal membrane much more accessible [41]. Lysobiphosphatidic acid (LBPA) interacts directly with ESCRT-III to induce its recruitment, skipping the traditional ESCRT process. However, in normal cells (non-tumor), such as dendritic cells, ALIX silencing enhanced MHC-II exosomal production but decreased CD63 expression in exosomes [36].

A comprehensive RNA interference screen also discovered that changes in the ESCRT machinery could lead to EV heterogeneity in size and content. HeLa-CIITA-OVA and dendritic cells were used in the study by Colombo et al. to investigate the effects of various factors on the secretion of EXOs (100,000 g pellets) (DCs) [36]. They found that silencing genes for ESCRT-0, HRS, STAM1, and ESCRT-1 all led to a reduction in the amount of exosomal protein secreted. According to the findings of Menck and colleagues, inhibitors of balanced Neutral sphingomyelinase (NSMase) are able to prevent the exosomal secretion that occurs in cells (as well as known as SMPD2 and SMPD3) [42] and they found that overproducing nSMase increases the exosome synthesis.

## Exosomes as drug carriers

Using nanocarriers frequently results in improved pharmacokinetics, safety, and bioavailability profiles for entrapped compounds. Many nanoparticle forms have been confirmed by the FDA, 1995 (Doxil), 1996 (onivyde), and 2005 (Abraxane) or have progressed to be studied in clinical-grade [43–45]. Typically, these nanoparticles are produced utilizing lipids or polymers because those substances give substantial protection against breakdown by serum nucleases and proteases. Exosomes act as nanovesicles that carry cargo for intercellular communication. However, as a result of their function in tumor formation and suppression of anti-tumor activity, exosomes from cancer cells can influence a wide range of intercellular processes. For example, circulating tumor-derived exosomes circFARSA promotes NSCLC metastasis by stimulating M2 macrophage polarization via the PTEN/PI3K/AKT pathway [46]. Interestingly, targeting exosomes in different diseases allows us to regulate the progression and spread of some diseases, such as cancer [47–49].

**Fig. 3:**
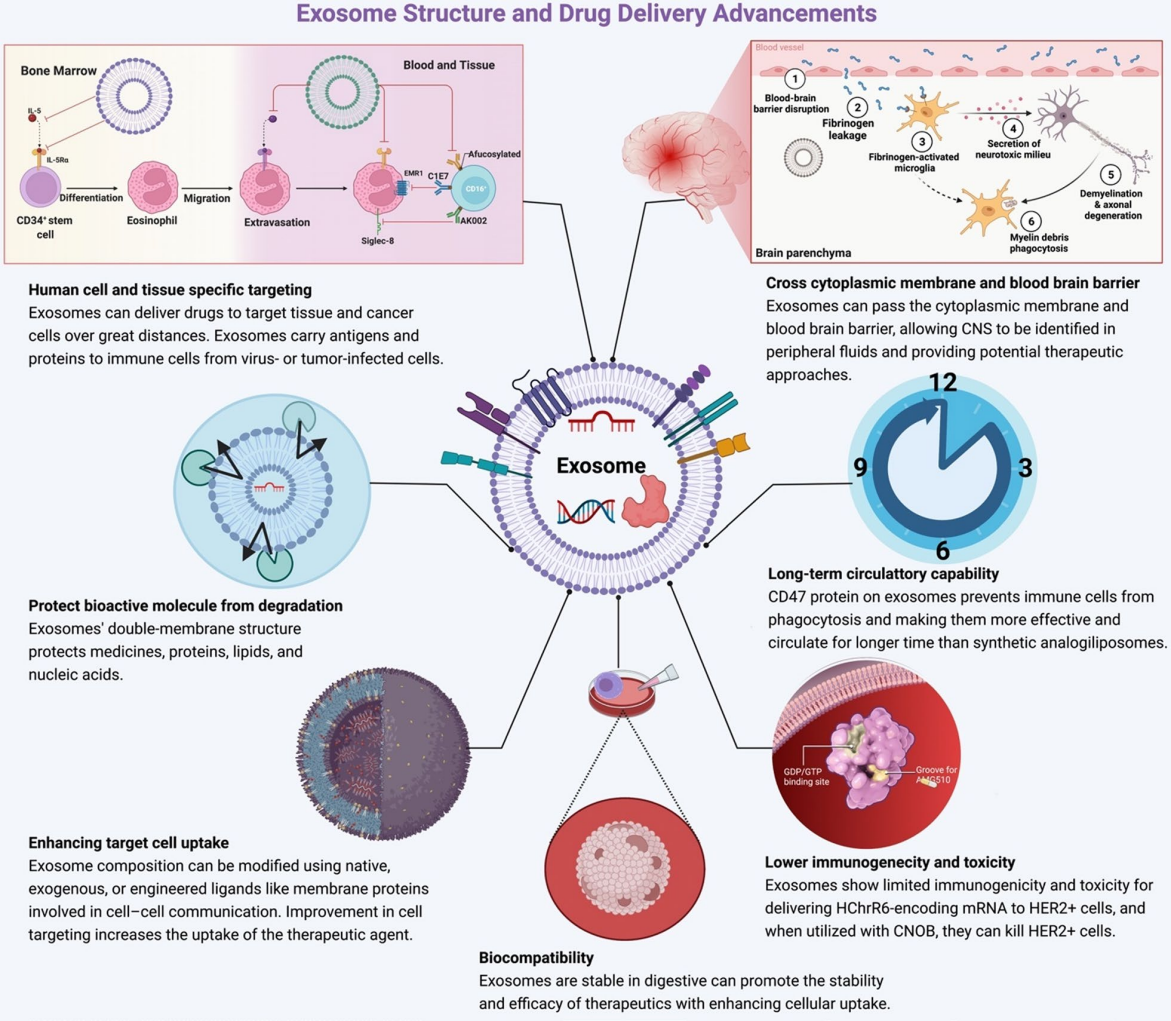
The preference of exosomes to be used as a suitable drug carrier technology. Characteristics like as biocompatibility, precise targeting, and sustained circulatory capacity make these nanomaterials appropriate for delivery. Furthermore, it has become a top candidate for drug and bioactive molecule transport due to its high selectivity, low immunogenicity, and low toxicity; it can cross the cytoplasmic membrane and the blood brain barrier

Exosomes, as natural transporters, provide a considerable benefit to use as a carrier in cancer therapies because their membrane is adorned with a variety of ligands and has long stabilities, long half-life [50], cross the cytoplasmic membrane, and blood brain-barrier that can be advantageous to target a specific tumor [51] (Fig. 3).

Recent studies have led to new ideas for treating cancer with therapeutic delivery systems based on exosomes made from cells (Table 1). Exosomes lack toxicity and immunogenicity, are promising as carriers of cytotoxic drugs including docetaxel, doxorubicin, and paclitaxel, and have better stability and tumor targeting [52–54]. Currently, a dual-functional exosome-based superparamagnetic nanoparticle cluster (SMNC-EXO) [55] has been designed, employing several superparamagnetic nanoparticles loaded to a single exosome to create a cluster. Thus, with external magnetic fields, SMNCEXOs have a potent capacity to transport therapeutic molecules to cancer cells [56]. Through molecular engineering, the expression of specific ligands can also be increased, and it was found that some forms of exosomes are better at delivering drugs than commonly used nanocarriers [57, 58]. This makes them attractive candidates for delivering cancer treatments.

**Table 1:** Exosomes as treatment carriers for different types of cancer

Exosome cargo	Donor	Cancer type	Drug loading method	Outcome/in vitro	Outcome/in vivo	Refs.
*Delivery of miRNAs*						
Let-7a miRNA	Fetal renal cells	Breast cancer	Transfection	–	Progression of tumor decreased	[59]
Let-7a miRNA	HEK293	Breast cancer	Transfection	–	Progression of tumor decreased	[59]
Suicide mRNA	HEK293T	Schwannoma tumors	Pre-transfected parent cells	–	Progression of tumor decreased	[60]
miR-335 − 5p	Stellate cell	Liver cancer	–	Liver cells progression and invasion decreased	Progression of tumor decreased	[61]
miR-379	MSC	Breast cancer	–	–	Death of tumor increased	[62]
miR-145 − 5p	MSC	Pancreatic ductalAdenocarcinoma	–	Propagation of PDAC cells and invasion decreased	Death of tumor increased	[63]
miR-25 − 3p inhibitor	Colorectal cancer cell	Colorectal cancer	–	Tube building of HUVEC cell decrease	Producing pre-metastatichousing for deceased	[64]
miR-146b	MSC	Glioma	Transfection	Propagation in cells of glioma decreased	Progression of tumor decreased	[65]
*Delivery of other RNAs*						
Cas9 mRNA	RBC	Breast cancer	–	Propagation of breast cancer cells decreased	Progression of tumor decreased	[66]
PTEN mRNA	Mesenchymal stem cell	Glioma	–	Propagation of glioma cells decreased	The size of the tumor decreased	[67]
ECRG4 mRNA	Serum	Tongue carcinoma	–	Propagation in cells of tongue carcinoma decreased	–	[68]
Hsp27 siRNA	Neuroblastoma cell	Neuroblastoma cell	–	Neuroblastoma cell differentiation decreased	–	[69]
KrasG12D siRNA	Fibroblast-like mesenchymal cells	Pancreatic Cancer	–	Panc-1 cell death increased	The size of the tumor decreased	[70]
*Delivery of proteins*						
MHC-I/peptide complexes	DCs	Breast cancer	–	The activity of T cells increased	–	[71]
Hsp70	Myeloma cell	Myeloma	–	The activity of T cells increased	The activity of T cells increased	[72]
Trial	Myeloid leukemia cell	Lymphoma	–	Leukemia cell death increased	Tumor development was not influenced	[73]
EGFR nanobodies	Neuroma cell	Epidermal	–	Propagation of epidermal carcinoma cells decreased	–	[74]
Competitive antagonist (SIRPα)	Fetal renal cell	Colon cancer	–	Macrophage ability to phagocytosis increased	Increased ability ofmacrophages for phagocytosis	[75]
*Delivery of chemical drugs*						
Doxorubicin	Breast cancer	Dendritic cell	Electroporation	Cells of breast cancer proliferation decreased	Progression of tumor decreased	[76]
Cisplatin	Hepatocarcinoma cell and ovarian cancer cell	Ovarian cancer and Hepatocarcinoma	–	Cells propagation in ovarian cancer andhepatocarcinoma decreased	Progression of tumor decreased	[77]
Paclitaxel	Macrophage	Lewis Lung carcinoma	Incubation	Cells in Lewis lung carcinoma propagation decreased	Progression of tumor decreased	[78]
Paclitaxel	Prostate cancer cell	Prostate cancer	Incubation	Cells in prostate cancer propagation decreased	–	[58]
Curcumin	Pancreatic cancer cell	Pancreatic cancer	Incubation	Cell death in pancreatic cancer increased	–	[79]
DOX	Immature DC	Breast cancer	Electroporation	Cells in breast cancer propagation decreased	Progression of tumor decreased	[80]
Imatinib	CML cell	Breast cancer	–	–	Progression of tumor decreased	[81]
5-FU	Schwannoma cells	Schwannoma tumor	Electroporation	Cells propagation in Schwannoma decreased	Progression of tumor decreased	[60]

## Exosomes and clinical trials as anticancer delivery

**Fig. 4:**
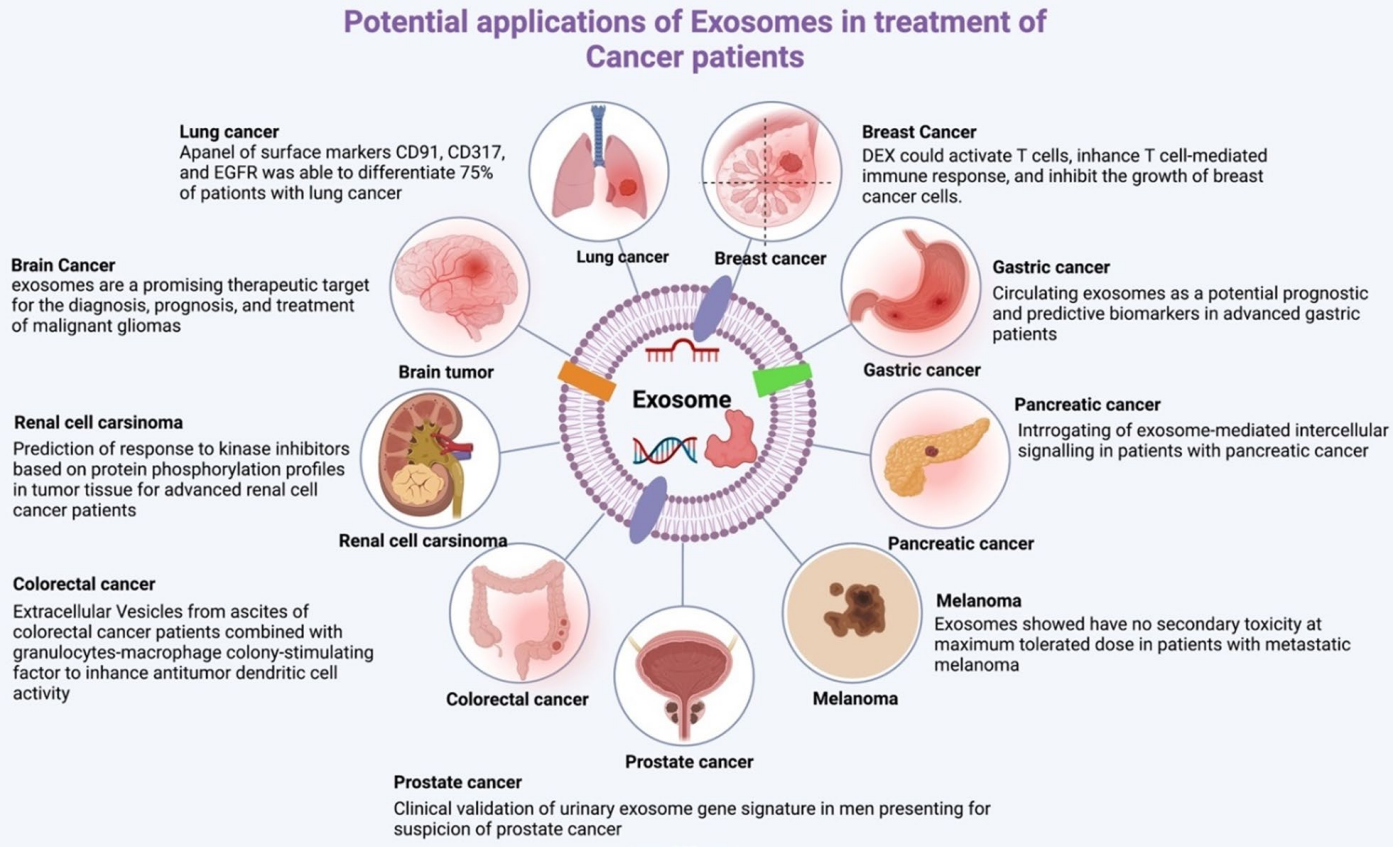
Exosome-mediated treatment has the potential to cure cancer disease. The different types of cancer that exosome-mediated technology is now used to treat are rising daily. The exosome-mediated treatment technology has been used to generate many cancer-based models for various significant human cancers, such as glioma, breast cancer, lung cancer, gastric cancer, pancreatic cancer, renal cancer, colorectal cancer, prostate cancer, melanoma, and other types of cancers, according to data from clinical trials released recently

Exosomes are currently being investigated as a potential tool and widely used for the delivery new class of medicinal drugs for cancer therapy in several clinical studies due to their favorable qualities, including their greater capacity to target cancer cells and their high integrity profile [82] (Fig. 4). They can deliver drugs directly into cells, which are difficult to reach with traditional delivery systems [83]. It is possible to transfect siRNAs into exosomes to transport them to the cells and tissues of interest. Because CD47 and other endogenous signaling ligands are expressed on the surface of exosomes, the half-life can be prolonged by significantly reducing MPS release and increasing cellular uptake.

Two types of exosomes are used in clinical trials: those produced from plants and those derived from human cells. In comparison, human exosome clinical studies are in the advanced stage, whereas plant exosomes are just in the early initial phases, and no patients have yet been enrolled in clinical studies. Because of their vesicle structure, exosomes have also been used as drug carriers in clinical trials (Table 2).

**Table 2:** Exosomes have been used as carriers for cancer diagnostics and therapeutics in clinical trials

Donor cell origin	Therapeutic agent	Targeting site	Phase (stage)	No. enrolled/patient	Administration	Status	Results	Number of clinical trials
Mesenchymal stromal cell-derived exosomes with KRASG12D siRNA	KRAS G12D siRNA	Metastatic pancreatic cancer	Phase 1	(n = 28)	–	Recruiting	Ongoing	(NCT03608631) [87]
Tumor Cell-derived Microparticles	Packing microparticles	Malignant ascites and pleural effusion	Phase 2	(n = 30)	Unknown	Chemotherapeutic drugs packaged into microparticles destroyed tumor cells effectively without any adverse effects from chemotherapy	Chemotherapeutic drugs packaged into microparticles destroyed tumor cells effectively without any adverse effects of chemotherapy	(NCT01854866)
Malignant pleural effusion	Tumor-derived microparticle, cisplatin	Malignant pleural effusion	Phase 2	(n = 90)	Unknown	Chemotherapy drugs were effectively encapsulated by microparticles and proved that this therapy could block tumor progression at the cellular and animal level	Chemotherapy drugs were effectively encapsulated by microparticles and proved that this therapy can block tumor progression at cellular and animal level	(NCT02657460)
Fruit-derived	Curcumin, curcumin conjugated with plant exosomes	Colon cancer	Phase 1	(n = 35)	Recruiting	Ongoing	Ongoing	(NCT01294072)
Grape derived	Grape extract, Fentanyl patch, mouthwash	Head and neck cancer, oral mucositis	Phase 1	(n = 60)	Active, not recruiting	Ongoing	Ongoing	(NCT01668849)
Cancer tissue and blood-derived	–	Pancreatic cancer	Pre-clinical phase applicable	(n = 111)	Recruiting	–	–	(NCT02393703)
Human bone marrow-MSCs	UNEX-42	Bronchopulmonary dysplasia	Phase 1	(n = 18)	Terminated	Ongoing	Ongoing	(NCT03857841)
HPV-OPSCC	–	Oropharyngeal cancer	Pre-clinical phase	(n = 30)	Recruiting	Ongoing	Ongoing	(NCT02147418)
Blood plasma-derived	–	Lung cancer	Pre-clinical phase	(n = 470)	Active, not recruiting	–	–	(NCT04529915)
Plasma exosomes	PD-L1 mRNA in plasma exosomes (pExo)	Non-small cell lung cancer	Pre-clinical phase	(n = 60)	unknown	Five kinds of radiation division detected different rate expressions of PD-L1 in pExo after 24 h, and 48 h of each stage of radiotherapy	Five kinds of radiation-division detected different rate expression of PD-L1 in pExo after 24 h, 48 h of each stage of radiotherapy	(NCT02869685)
Metastatic Meningitis	–	Breast cancer	Not applicable	(n = 30)	Not yet recruiting	–	Appropriate gained data lets a promising strategies to progress metastatic cancer meningitis	(NCT05286684)
Non-small cell lung cancer in its early stages	–	Lung cancer, non-small cell lung cancer	Not applicable	(n = 30)	Recruiting	–	–	(NCT04939324)
Ovarian cancer with a high grade	Sequencing of miRNA/lncRNA	Ovarian cancer, high-grade serous carcinoma	Not applicable	(n = 160)	Unknown	–	Ongoing	(NCT03738319)
Prostate cancer cells	Exointelliscore prostate	Prostate cancer	Not applicable	(n = 2000)	Completed	–	RP patients with a high EPI score were found to have a lower likelihood of developing low-risk pathology, which could have important implications for AS decisions	(NCT02702856)[88]
Pancreatic ductal adenocarcinoma (PDAC)cells	–	Pancreatic ductal adenocarcinoma	Not applicable	(n = 52)	Completed	–	–	(NCT03032913)

Furthermore, exosomes are obtained from three primary sources in clinical trials: DCs, MSCs, and tumor cells from patients. Purified exosomes can be obtained via ultrafiltration (UF) or differential centrifugation (DC) and ultracentrifugation (UC) on sucrose. Alternatively, exosomes may include tumor antigens to promote anti-tumor immunity in a patient or anticancer drugs to trigger cytotoxicity in cancer therapy. For cancer treatment, exosomes that transport chemo drugs or siRNA have been employed in combination with tumor antigens.

Cancer therapy, such as oncogene inhibition, may use several approaches based on exosomes. For example, exosomes derived from mesenchymal stem cells can be used to treat pancreatic cancer patients with the presence of the KrasG12D mutation, such as in a phase I trial (NCT03608631) sponsored by the M.D. Anderson Cancer Center (Texas, USA), where patients are injected with KrasG12D-targeted siRNA-loaded exosomes, thereby reducing the oncogenic KRAS gene expression in pancreatic tumors [84]. The immunotherapy strategy was also tested in a clinical trial (NCT01159288) for patients with unresectable NSCLC using dendritic cell-derived exosomes loaded with tumor antigens [26]. There was no particular T cell response to cancer cells expressing the antigen of interest; however, some patients significantly increased NK cell activity. An essential and critical endpoint was not fulfilled, and the trial had to be ended. Due to the specificity of their tropism and capacity to trigger a specific type of inflammatory response, tumor cells are an excellent source of exosomes for cancer therapy. Furthermore, an antisense drug targeting the tyrosine kinase cell surface receptors of the tumor was employed to prevent tumorigenesis in a phase I trial (NCT01550523) using autologous glioma cells pretreated with insulin-like growth factor I receptor (ILF1R) [85].

Moreover, methotrexate (MTX) and cisplatin were the anticancer drugs tested in the NCT01854866 preclinical and clinical trials based on the exosomal approach. In preclinical experiments, the survival rate was more remarkable when MTX was used as the anticancer drug [86]. In the NCT02657460 trial, MTX was used as the encapsulated anticancer therapy, while cisplatin was used as a comparison drug. Furthermore, patients with metastatic pancreas cancer are being treated with KRASG12D siRNA and exosomes produced from mesenchymal stromal cells in clinical trial NCT03608631, both of which have been promoted as additional anticancer drug categories. Depending on the outcomes of the clinical trials described above, exosomes may have therapeutic applications for cancer.

## Exosomes in cancer therapy: challenges and strategies to overcome

Although exosome as a carrier for cancer therapy has a bright future fingerprint and is a promising approach, there are still some outstanding obstacles and challenges that make it difficult to use in clinical trials because it is a recent discovery and has not been clinically tested.

**Fig. 5:**
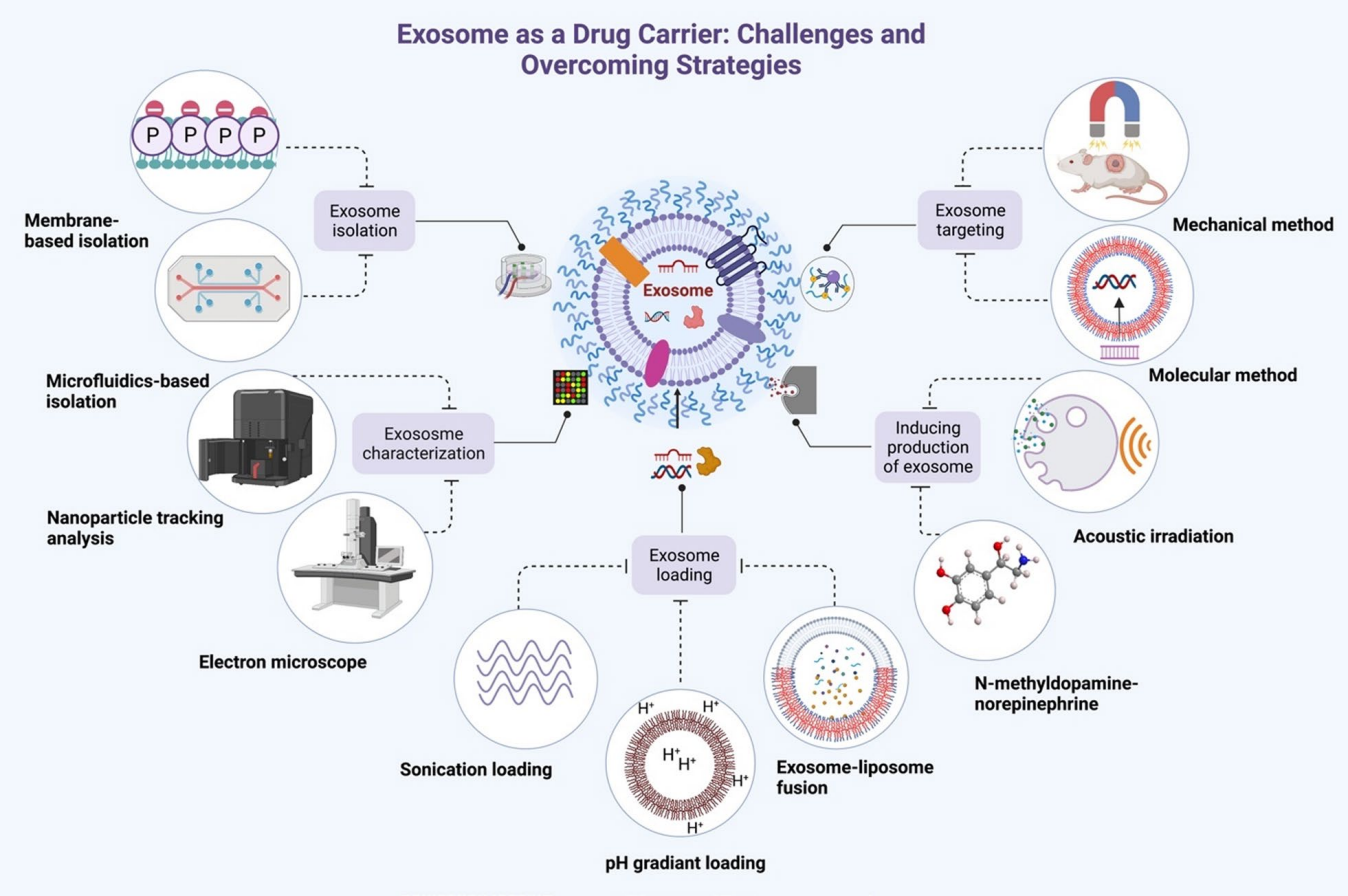
Illustrates the main challenges of using exosomes as a therapeutic carrier and the strategies to overcome these challenges. Traditional therapeutic carriers, which are inadequate for transporting drugs for the treatment of cancer, could be replaced by exosomes

When it comes to the potential use of exosomes as drug carriers in combination with a variety of cutting-edge strategies, the most pressing issues include exosome purification insufficiencies, poor characterization, loading efficiency, tumor targeting, and the production of exosomes by recipient cells [89] (Fig. 5).

### Isolation of exosomes

Despite the rapid development of exosome studies, the separation and purification methods remain underdeveloped and unstandardized [90–92]. An efficient exosome isolation approach should be capable of removing exosomes from various sample matrices. However, separating exosomes from net biological liquids is complicated because several biological fluid contents, such as lipoprotein, chylomicrons, and microvesicles, interfere with the size of exosomes (30–150 nm) [93, 94]. In addition, several of these extracellular vesicles have identical physical qualities to exosomes, for instance, the size and density of these EVs, making separating exosomes challenging [95].

It has become increasingly possible to isolate exosomes in large quantities and with high purity in response to rapid advances in science and technology. In order to facilitate the isolation of exosomes, many properties of exosomes, such as density, shape, size, and surface proteins, are taken advantage of. There are three types of separation techniques: conventional methods, microfluidics-based methods, and membrane-based separation methods. However, traditional practices like ultrafiltration, ultracentrifugation, size exclusion chromatography, immunoaffinity, and polymer-based precipitation are well-founded and openly utilized. However, these conventional strategies have not been found to be useful or efficient [96].

#### Strategies to overcome exosome isolation

Several novel strategies proved their effectiveness based on emerging exosome isolation methods. They helped a step forward using isolated and purified efficient exosomes as a drug delivery carrier in cancer therapy (Table 3).

**Table 3:** Emerging strategies to overcome exosome isolation challenges

Strategy	Classification basis	Method	Principle	Technique	Advanced features	Refs.
Membrane-based separation	Magnetizing of oxide metal	Micron-sized metal oxide	Phosphate groups of exosomal lipid bilayer membrane can bind especially with particular oxide of metals (e.g., TiO2, ZrO2)	TiO2-based separation	High yield and fast technique	[98]
		Micron-sized magnetic metal oxide		Magnetic TiO2-based separation	High efficiency to uptake urine-derived exosomes and its fast technique	[98, 99]
Microfluidics-based isolation	Physical properties-based microfluidics	Active isolation	External forces affect different physical characteristics of nanoparticles, such as size, electrical parts, and density	Acoustic force	Sufficient isolation efficacy, fast, versatility, and biocompatibility,	[101, 107]
				Centrifugal microfluidics	Low centrifugal force, comparable recovery, and quick	[103]
		Passive isolation	Combined platforms depended on intricate channel makeup or hydrodynamic properties	Pillar-based microfluidics	Samples with a high pixel density	
				Hydrodynamic-based microfluidics	Less complexity of fabrication and operation, fast, isolation of exosomes directly from entire blood, reproducibility, and portable	[108]
	Immunoaffinity-based microfluidics	Mobile-coated medium	Magnetic beads or other magnetic nanoparticles coated with antibodies, together they have an enormous surface area and greater handling flexibility	Magnetic bead microfluidics	Sufficient separation, separation performed from entire blood	[105]
				Merged Raman chip microfluidics	Significant targeting, production, and sensitive	[100]
		Stationary-coated medium	Depend primarily on reaction exosomes with antibodies/aptamers fixed on the roof of microchannels	Double-use chip activated with antibodies	Specificity, high flow rate, high separation efficiency	[109]
				Antibody-functionalized enhanced lipid membrane microarrays	High grade of susceptibility and sensitivity	[110]

##### Membrane-based isolation strategy

**Fig. 6:**
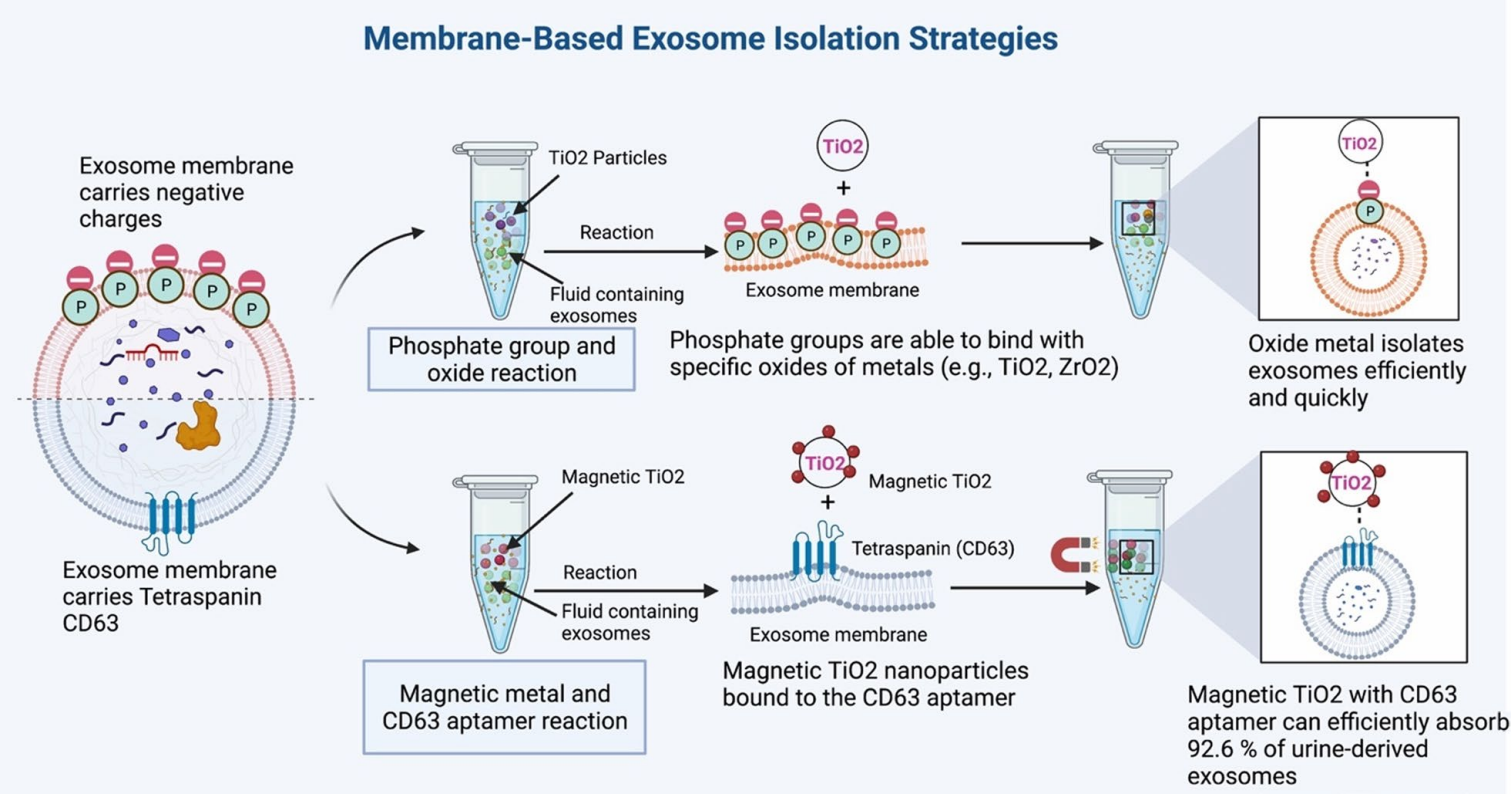
An effective strategy to overcome the exosome isolation challenge is the membrane-based strategy, which uses membrane properties of exosomes that have a great potential to capture exosomes and separate them from other kinds of nanoparticles

In membrane-based isolation, the presence of an abundance of phosphatidylserine as a negative charge on the membrane of exosomes facilitates the design of a number of innovative strategies [97]. Furthermore, the majority of the exosomal lipid bilayer membrane is made up of amphiphilic phospholipids, which form the hydrophilic phosphate head on the membrane's surface [98]. Exosomes are arranged in this manner because amphiphilic phospholipids are more abundant. Because of this distinguishing feature, phosphate groups can bind particularly well with specific oxides of metals (e.g., TiO2, ZrO2) (Fig. 6). Significantly, Gao and his colleagues recovered exosomes by combining micron-sized TiO2 molecules with the phosphate groups located on the exosomal outer surface in a very affinitive manner [98]. The oxide metal strategy is capable of isolating exosomes quickly and with high levels of efficiency in a short period. For example, Zhang et al. found that magnetic TiO2 nanoparticles bound to the CD63 aptamer could efficiently absorb 92.6% of urine-derived exosomes in a short period [99].

##### Microfluidics-based isolation strategy

Microfluidics devices paired with external force, such as electrical, acoustic, and magnetic fields, are increasingly effective strategies. Microfluidic devices depend on physical properties, typically membranes with nanopores, nanofilters, microvillus, an acoustic field, and an electric field for refining exosomes, depending on the physical characteristics, and are categorized into two classes; active and passive separation techniques [100]. Acoustic technique, dependent on an external acoustic force, is a dynamic technique used in effective separation [101]. Another active technique, the electrical method, is dependent on the strength of an electrical field, particle size, and electrical characteristics [102]. Finally, the centrifuge microfluidics approach uses double filtration compartments (the first with 600 nm and the second with a 20 nm pore size) [103] to catch non-exosomal particles and isolate exosomes.

Microfluidics devices have also used platforms based on complex channel design or hydrodynamic features in the passive separation technique. For example, using an inertial-based microfluidic method, Tay and colleagues have isolated exosomes and nanoparticles from whole blood [104].

Immunoaffinity microfluidics techniques are promising alternatives to physical properties-based microfluidics for exosome isolation. These strategies use the interaction between antigen and antibody to isolate targeted exosomes, while immunoaffinity uptake can be accomplished with stationary and mobile antibody-coated mediums in most cases.

Furthermore, microfluidics-based exosome harvesting and secretion can be improved by using magnetic beads or other nanoparticles coated with antibodies in a mobile-coated medium, according to the findings of Sanco-Albero and colleagues and Wang et al., respectively. This platform may be able to isolate exosomes from whole blood or serum [105, 106].

### Characterization of exosomes

Traditionally, EVs have been classified based on their physical properties, such as particle size, membrane surface electrical charge and density, and biological properties, such as their internal and external biomolecular structure, such as surface-linked antigens [111, 112].

The therapeutic value of exosomes produced from multivesicular bodies increases when they are highly characterized. In contrast, the absence of exosome-specific characteristics due to the heterogeneity and size variance creates challenges in isolating high-quality standardized exosomes.

#### Strategies to overcome exosome characterization

**Fig. 7:**
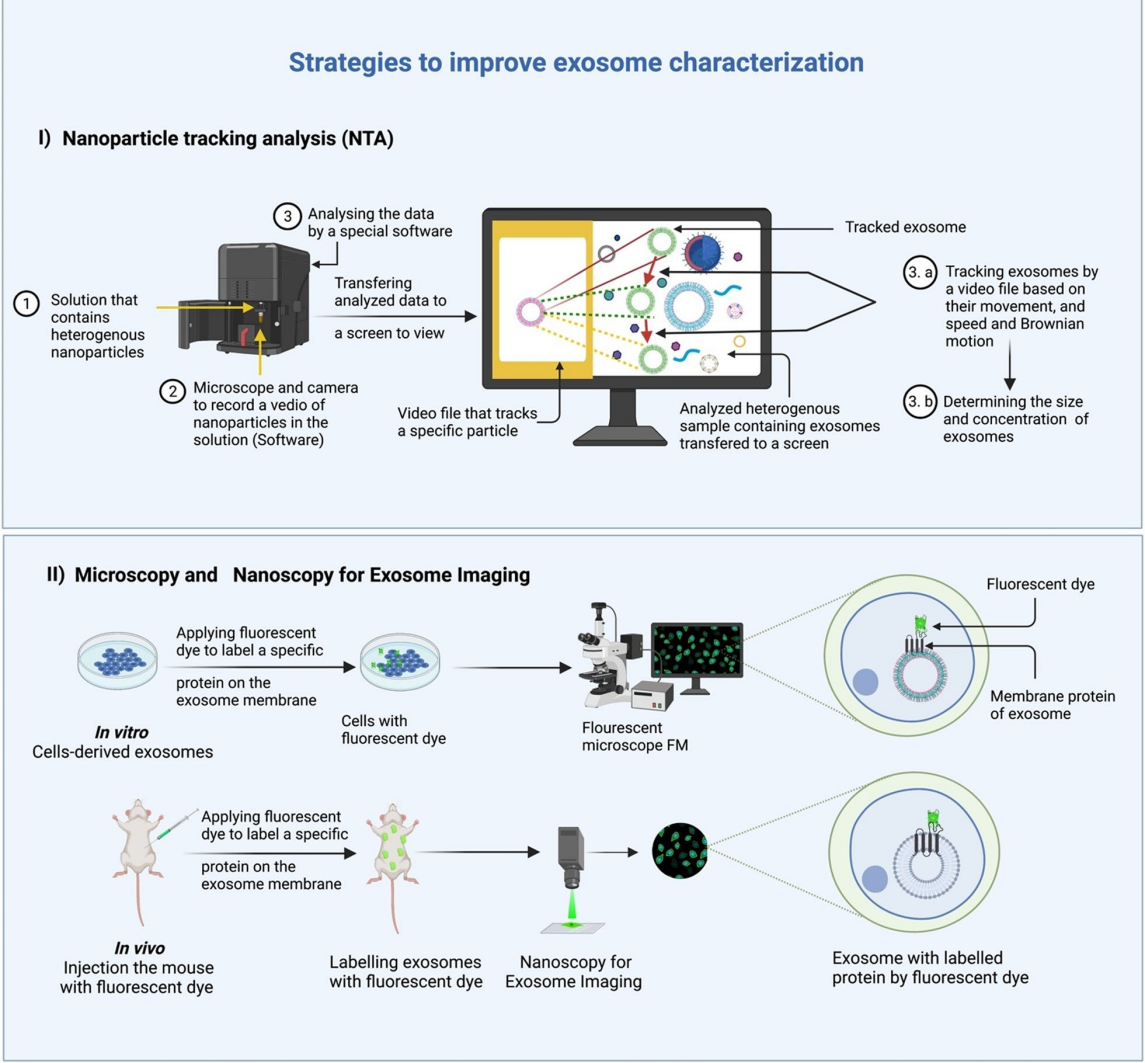
Characterization of exosome using two most advanced strategies: (i) nanoparticle tracking analysis NTA and (ii) fluorescent microscope FM. The developed NTA strategy allows exosomes to be tracked through a video file tracker and determine the speed and movement of the exosomes. At the same time, a fluorescent microscope FM can be used to study exosomes in vitro and in vivo by marking a specific protein on the membrane of an exosome with a fluorescent dye

Several strategies have been designed to address the exosome characterization limitations of heterogeneity and size variance. Here, we give an overview of the most important ones; (i) by using nanoparticles tracking analysis (NTA) to determine their size, and (ii) by using microscopy and nanoscopy for exosome imaging techniques to visualize the exosomes, followed by labeling, which helps in loading specific cargo for specific targeting, including the targeting of tumor cells (Fig. 7).

##### Nanoparticle tracking analysis (NTA)

Nanoparticle tracking analysis (NTA) uses identical physical characteristics to determine the size of nanoparticles. The software measures the particle’s size and concentration using a video file from a microscopic technology that tracks the movement of exosomes, and their speed and Brownian motion are monitored [113–115]. Furthermore, NTA’s recognition boundary is based on the particle's visibility and, by extension the microscope's resolution. Because NTA can track several particles at once, it can be used to identify samples that have been scattered [116, 117]. The NAT methodology is similar to Dynamic Light Scattering (DLS) methods, which determine a particle's hydrodynamic radius based on variations in laser transition caused by Brownian motion [113]. On the other hand, NTA is a more credible and dependable technology than DLS because DLS can characterize the diameter of particles ranging from 1 nm to 6 m and is only accurate with particles in homogeneous solutions [118, 119].

##### Microscopy and nanoscopy for exosome imaging

Fluorescent microscopy (FM) developments have made it possible to directly image in vivo and in vitro exosomes without harming them. This is another practical way to get around the limitations of characterizing exosomes. FM allows multiple fluorescent dyes to stain and mark cellular components simultaneously [120]. Exosomes are usually observed by directly keeping specific surface proteins with fluorescent dyes or transfecting fluorescent fusion proteins into the target cell's cytoplasm. Fluorescent proteins provide constant fluorescent signals and accurate labeling [121, 122]. Recent progress in ultra-resolution imaging has opened new horizons to the study of exosomes. These include total internal reflection fluorescence microscopy (TIRF) and single-molecule localization microscopy (SMLM), which includes photoactivation localization microscopy (PALM) and stochastic optical reconstruction microscopy (STORM) [123–125]. The above studies provided conclusive evidence that it is possible to visualize exosomes with ultra-resolution techniques.

### Loading cargoes into exosome

Exosomes are possible medicinal carriers that can limit tumor development by incorporating drugs. However, knowledge regarding exosome contents and the loading mechanism is not well understood. The lack of an appropriate standardized loading strategy is the main challenge for bringing exosome innovation technology into clinics.

#### Strategies for loading cargoes into exosome

**Fig. 8:**
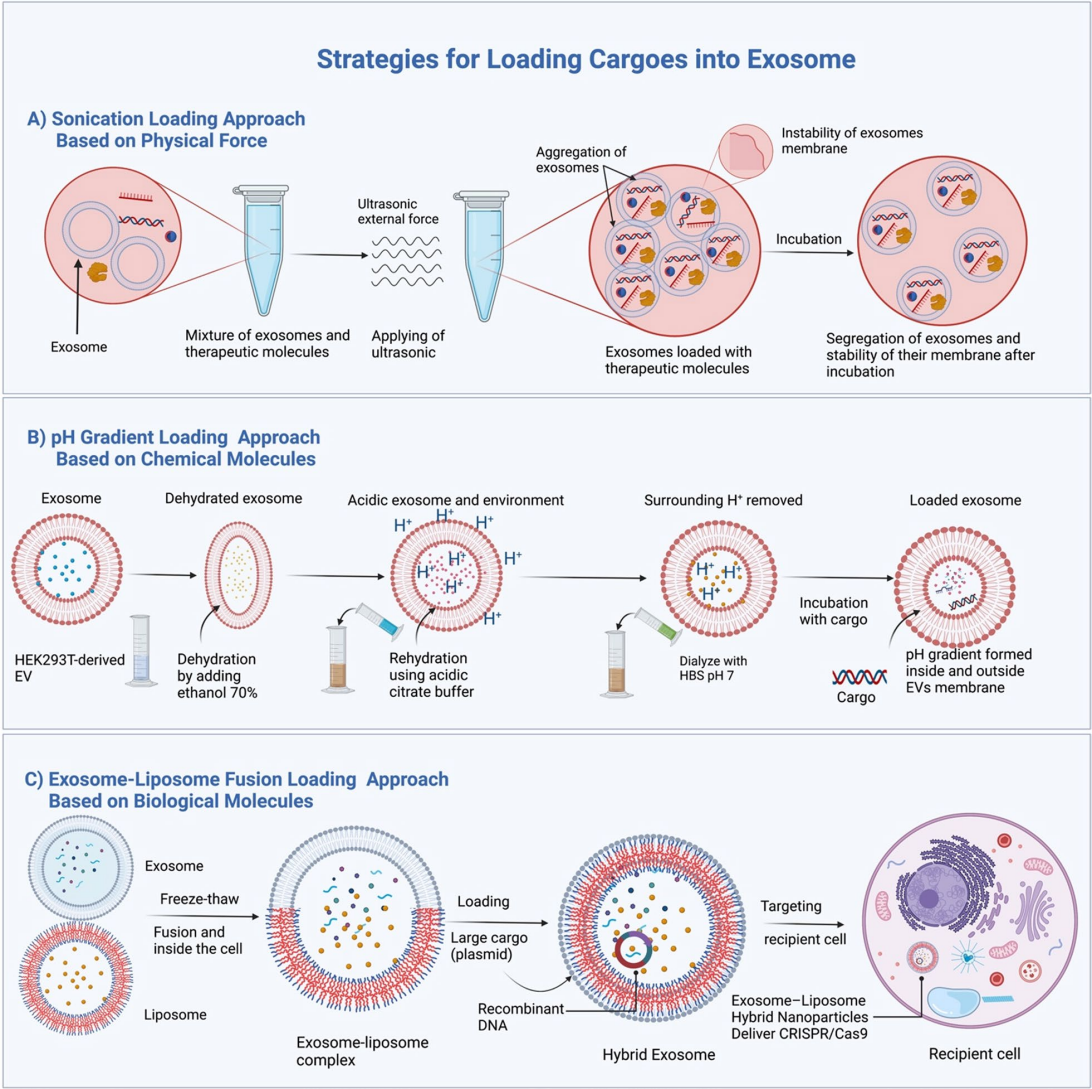
Loading exosomes with cargos based on physical, chemical, and biological techniques. **A** Sonication as an effective technique depends on the physical force to load bioactive molecules into exosomes. In contrast, **B** in the pH gradient technique, the chemical solutions (ethanol 70% for dehydration, citrate buffer rehydration, and HBS for dialyzing) are utilized to load bioactive molecules into exosomes. **C** In The exosome-liposome fusion technique, the biochemical properties of phospholipid of exosome and liposome are exploited to help merge exosome and liposome, which is significantly essential to load large and hydrophobic bioactive molecules into the exosome-liposome hybrid

Depending on the chemical composition of the packaged structures, the loading strategies for packaged molecules into exosomes and associated efficiencies vary. Here we discuss the three effective strategies for loading molecules into exosomes (Table 4): (i) sonication loading, (ii) Potential of hydrogen (pH) gradient loading, and (iii) exosome-liposome fusion loading (Fig. 8).

**Table 4:** Loading approaches of exosome-based on the newest advancement

Approaches	Principle	Loading efficiency	Cargo stability	Exosomes stability	Type of loaded cargo	Ability to load large cargo	Circulation period	Refs.
Sonication	Uses ultrasound with high frequency to transfer therapeutic cargos	Loading efficiency is high as showed that siRNA loaded into EV by sonication at a 325% increase than electroporation	Can keep the integrity of the cargo against deformation	Incubation can solve the instability of exosomes after exosome-liposome fusion	This technique can load chemicals, proteins, small nucleic acids, and nanomaterials	Sonication can load a wide range of bioactive molecules into exosomes, including small and large molecules	The circulation period is not affected by sonication since incubation can reform the temporarily generated pores in the exosome membrane	[151–154]
pH gradient	It depends on the change in the biochemical environment inside and outside the exosome	High loading efficiency as sonication approach	Nucleic acid (miRNA or mRNA) cargos can be protected and very stable when in a low pH environment	Exosomes in the ex vivo acidity environment appear stable	Its loading efficiency comparable to the sonication method	Able to load large cargos such as CRISPR/Cas9 vector	Remaining period increases because low pH allows more activity and stability	[155]
Exosome-liposome fusion	The principle is lipid-lipid fusion between exosome and liposome	High loading ability to hydrophobic and hydrophilic compounds	The fusion of exosome and liposome approach could protect cargo from the plasma and immune system and makes the cargo more stable	Increase the hybrid exosome stability more than exosome alone	Hydrophobic drugs and hydrophilic drugs	This approach provides a great opportunity to load large cargos such as CRISPR/Cas9 vector	Because of their endogenous nature, the hybrid exosomes (exosome-liposome complex) have a longer circulating period, which makes it considered an approach for loading cargos	[156–158]

##### Sonication loading approach

This method uses ultrasonic force to treat a mixture of exosomes and a drug [126]. The Sonication method has a high loading efficiency of exosomes. It can be used to load various biomolecules, including RNAs, mRNA, DNA, and proteins, as well as to load macromolecules [127–139]. In addition, the combination of sonication and incubation increases the stability of the exosome membrane and prevents the aggregation of the exosomes [140].

Furthermore, the sonication approach offers many benefits, including improved cytotoxicity, a high drug dosage loading efficiency, and prolonged drug release [141, 142].

##### pH gradient loading approach

This method has the same efficiency as ultrasound without affecting the stability of the cargo. This method uses a pH gradient to make the EVs more acidic. When the EVs are more acidic, negatively charged cargoes like nucleic acids are more likely to be loaded into these extracellular vesicles. By making EVs more acidic, microRNA (miRNA), small interfering RNA (siRNA), and single-stranded DNA (ssDNA) can be loaded into EVs more efficiently. This creates a pH gradient across their membranes, which can then be used to increase EV loading capacity [143]. According to a recent study, the uptake of EVs by cells and the cytotoxicity of EVs in mice are not affected by the pH gradient loading technique [143]. The procedure includes dehydration of EVs with 70% ethanol, rehydrated in acidic citrate buffer (pH 2.5), and then dialyzed against 1X HEPES-buffered saline (pH 7) to replace the acidic environment around them [144]. This made a pH gradient inside and outside the EVs membrane. Furthermore, experimental results showed that the optimal load parameter of the cargo is incubation at room temperature (22 °C) for 2 h at pH 2.5 [145].

##### Exosome-liposome fusion loading approach

The membrane fusion of exosomes and nano-liposomes is a unique and straightforward membrane-engineering approach for modifying the surface of exosomes through direct membrane fusion between synthetic liposomes and exosomes following their release from parent cells. This technology allows us to modify the surface features of exosomes to minimize their immunogenicity, increase their colloidal stability, and increase exosome half-life [146].

A liposomal targeting moiety such as peptides or antibodies, or polyethylene glycol (PEG) can be used to modify the exosome surface characteristics [147]. Likewise, the complex vehicles can increase the efficiency of encapsulating drugs and preserve the role of exosomes [148], which aids in increasing the half-life of the complex vehicles in circulation [149].

Interestingly, Lin et al. discovered that hybrid exosomes-liposomes could package big-size plasmids, for instance, the expression of CRISPR-Cas9 vectors, more efficient than the exosome alone. Moreover, these fused exosome-liposome vesicles could be entered into MSCs and express the loaded genes, presenting a promising opportunity for in vivo gene modification [150].

### Quantities of exosome

Exosomes are extracellular vesicles that are very small in size and are often isolated in deficient amounts. This low yield has been a barrier to advancing fundamental science related to exosome analysis and applications in the delivery of drugs [159].

#### Strategies to increase production of exosome

Another main concern about using exosomes as a carrier in therapeutic cancer is that they are less or insufficient for clinical applications. Various strategies have been developed to bypass this limitation in order to produce enough exosomes. The most developed strategies include upregulating the six-transmembrane epithelial antigen of prostate 3 (STEAP3), syndecan4, and NadB. The expression of these genes together helped yield exosomes in a very high quantity. In addition, exosomal mRNA expression was boosted by around 15–40 folds due to the application of EXOtic devices (EXOsomal Transfer Into Cells) by Kojima and colleagues [160]. Similarly, A recent study showed that when N-methyldopamine and norepinephrine are used with small molecule modulators, MSCs may make three times as many exosomes as they would without these small molecule modulators [161]. This is another excellent method for increasing the production of exosomes. Finally, promoting or overexpressing some biomolecules can be a promising strategy to increase the exosome yield. For example, enhancing hypoxia in MSCs, overexpressing of tetraspanin CD9 in HEK293, or overexpressing of hypoxia-inducible factor-1α in MSCs can increase the exosome production by 1.3 fold, 2.4 fold, and 2.2 fold, respectively [162–164].

### Tumor targeting of exosomes

Natural transporters, exosomes, offer a considerable advantage in cancer therapy since the surface of exosomes is coated by various molecules that can be used to target tumors more effectively. In vitro accumulation experiments have revealed that tumor cell-derived exosomes can be targeted homogeneously [165]. Nevertheless, the targeting of the tumor varies considerably from one study to the next in vivo. Smyth et al. found that exosomes secreted by 4T1, MCF-7, and PC3 cells showed minimal tumor accumulation after being injected intravenously [166]. In vivo dextran sulfate inhibition of scavenger receptor-A (SR-A) impaired monocyte/macrophage-mediated hepatic clearance of exosomes in mice, resulting in a fivefold increase in tumor exosome accumulation [167]. Based on these findings, it appears that exosomes will need to be optimized in order to achieve effective tumor targeting.

#### Strategies to improve tumor targeting exosome

**Fig. 9:**
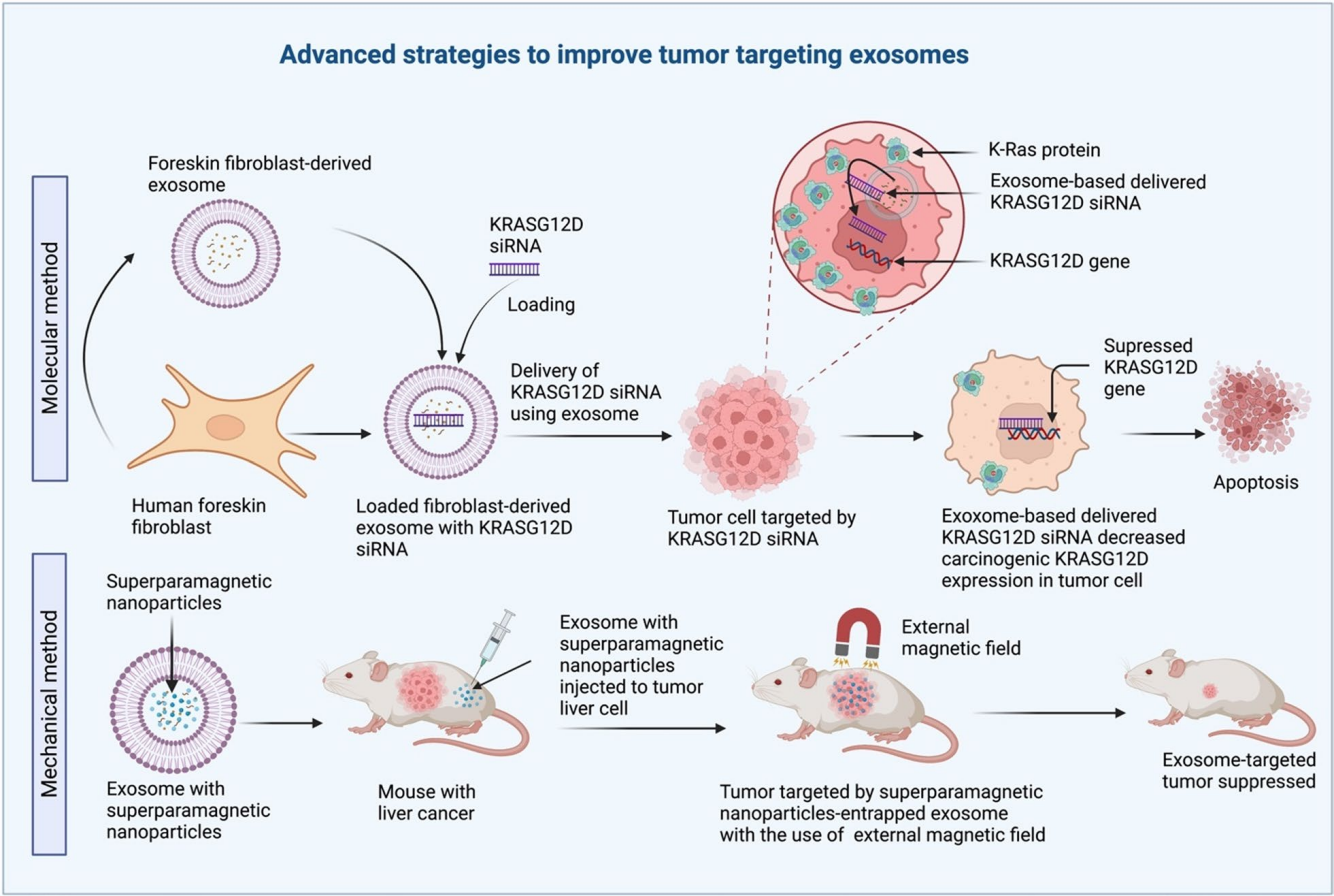
In the mechanical approach, Exosomes that contain superparamagnetic nanoparticles can be used to target tumors with an external magnetic force; just like the molecular method, exosomes can carry KRASG12D siRNA to tumor cells to lower KRASG12D expression. These are two innovative approaches for treating target malignancies that rely on exosomes as the delivery vehicle

Improving the capacity of nanovesicles for cancer therapy is a new area of intense research, and various strategies have been developed to solve this issue in order to improve tumor targeting exosomes. The most developed strategies include: (i) the molecular method, and (ii) the mechanical method, which have been shown to be more precise and effective than the more common methods (Fig. 9).

##### Molecular methods improve tumor targeting exosome

The molecular technique is built on the foundation of predictive biomarker molecules and a favorable protein expression profile. Kamerkar and his colleagues recently reported that exosomes from normal human foreskin fibroblasts could effectively carry KrasG12D siRNA to pancreatic tumor cells in vivo [84]. In a mouse model of pancreatic cancer, this decreased the amount of cancer-causing KrasG12D, inhibited tumor cell spreading, and an elevated total alive [84]. Furthermore, it has been shown that exosomes made from fibroblasts have the right protein expression profile on their membrane, which helps them target tumors effectively.

Interestingly, to better target a certain type of cancer, some researchers have molecularly altered exosome-producing cells to produce more ligands on the surface of the exosomes than naturally occur. For example, exosomes with Lamp2b-IL-3 have been used to target chronic myeloid leukemia (CML). This is because CML cells overexpress IL-3 receptors, which inhibit CML cells from growing in vivo and in vitro [168].

##### Mechanical methods improve tumor targeting exosome

In addition to molecular methods, mechanical methods that use superparamagnetic nanoparticles to trap exosomes and a magnetic field at the tumor site have also been developed to improve tumor targeting. Qi and his team were able to use superparamagnetic exosomes to deliver doxorubicin and slow down the growth of tumors in a subcutaneous animal model of liver cancer [169]. The increasing capacity of exosomes to target specific tumors has given a fresh perspective of life, resulting in increased demand for an innovative approach made possible by a novel method.

## Conclusion

EVs are increasingly considered key mediators of intercellular communication due to their ability to deliver various chemicals and carry signals for long distances. The ability of EVs to alter the immune system’s functioning indicates that they could be exploited as a cell-free therapeutic approach for a variety of diseases. EV-based therapies against different kinds of cancers have shown promise in a number of studies. However, before the medical promise of exosomes as drug carriers can be fully realized, some main challenges need to be addressed. First, exosome isolation has always been one of the most formidable problems in exosome-based drug delivery. Importantly, exosome loading and even cell targeting efficiencies are currently low for some drugs, especially hydrophobic drugs, so higher exosome isolation efficiency is needed to compensate. There should be more research attempts to improve exosome isolation, characterization, loading targeting, and production to ensure cell and tumor targeting specificity.

## Data Availability

The analyzed data sets generated during the study are available from the corresponding author on reasonable request.

## Abbreviations

CTLs: Cytotoxic T lymphocyte
CML: Chronic myeloid leukemia
EVs: Extracellular vesicles
ESCRT: Endosomal sorting complex required for transportation
EPI: ExoDx prostate intelliscore
EGFR: Epidermal growth factor receptor
DCs: Dendritic cells
DEXs: Dendritic exosomes
HSPGs: Heparin sulfate proteoglycans
HeLa cells: Henrietta lacks
MHC: Major histocompatibility complex
MVB: Multivesicular body
MSCs: Mesenchymal stem cells
Mps: Membrane-associated periodic signaling
ILVs: Intraluminal vesicles
NK cells: Natural killer cells
n-SMase2: Neutral sphingomyelinase-2
PEG: Polyethylene glycol
SMNC-EXOs: Superparamagnetic nanoparticle cluster exosomes
STEAP3: Six-transmembrane epithelial antigen of prostate 3
SR-A: Scavenger receptor-A
TDEs: Tumor-derived exosomes
